# Tipping the Scale from Disorder to Alpha-helix: Folding of Amphiphilic Peptides in the Presence of Macroscopic and Molecular Interfaces

**DOI:** 10.1371/journal.pcbi.1004328

**Published:** 2015-08-21

**Authors:** Cahit Dalgicdir, Christoph Globisch, Christine Peter, Mehmet Sayar

**Affiliations:** 1 College of Engineering, Koç University, Istanbul, Turkey; 2 Theoretical Chemistry, University of Konstanz, Konstanz, Germany; Icahn School of Medicine at Mount Sinai, UNITED STATES

## Abstract

Secondary amphiphilicity is inherent to the secondary structural elements of proteins. By forming energetically favorable contacts with each other these amphiphilic building blocks give rise to the formation of a tertiary structure. Small proteins and peptides, on the other hand, are usually too short to form multiple structural elements and cannot stabilize them internally. Therefore, these molecules are often found to be structurally ambiguous up to the point of a large degree of intrinsic disorder in solution. Consequently, their conformational preference is particularly susceptible to environmental conditions such as pH, salts, or presence of interfaces. In this study we use molecular dynamics simulations to analyze the conformational behavior of two synthetic peptides, LKKLLKLLKKLLKL (LK) and EAALAEALAEALAE (EALA), with built-in secondary amphiphilicity upon forming an alpha-helix. We use these model peptides to systematically study their aggregation and the influence of macroscopic and molecular interfaces on their conformational preferences. We show that the peptides are neither random coils in bulk water nor fully formed alpha helices, but adopt multiple conformations and secondary structure elements with short lifetimes. These provide a basis for conformation-selection and population-shift upon environmental changes. Differences in these peptides’ response to macroscopic and molecular interfaces (presented by an aggregation partner) can be linked to their inherent alpha-helical tendencies in bulk water. We find that the peptides’ aggregation behavior is also strongly affected by presence or absence of an interface, and rather subtly depends on their surface charge and hydrophobicity.

## Author Summary

Small proteins and peptides have important biomedical implications with their ability to penetrate cells, disrupt cell membranes, or form—potentially neurotoxic—fibrillar aggregates. It is rather intriguing that the same peptide can take part in all these processes depending on its environment. Hence it is important to understand how the environment guides the peptide from a disordered state to an alpha-helix or beta-sheet. By studying two different peptides with computer simulations, we find that one clue to this puzzle is the state of the allegedly disordered molecule in water, which is in fact characterized by a vast number of partially folded short-lived conformations. We investigate how interaction with macroscopic interfaces affects this multi-state conformational ensemble by promoting an alpha-helix over the other conformers. Furthermore, we show that association of two peptides can also be viewed as an interaction with an interface—a molecular interface. We show that the impact of macroscopic and molecular interfaces strongly depends on their ability to enforce partitioning of hydrophobic and hydrophilic residues. The effect gets more subtle as soon as macroscopic partitioning is coupled with peptide association where two interfaces compete: the relative balance of opposing forces determines aggregate stability and structure.

## Introduction

The partitioning, folding and aggregation properties of soluble peptides and small proteins at hydrophobic/hydrophilic interfaces (HHI) play a crucial role in biomedically important processes. One example that has been extensively investigated over the last years are amyloid-fibril forming peptides which are responsible for many human disorders, among them severe neurodegenerative diseases [1–3]. The amphiphilic character of the involved amyloidogenic proteins and peptides, such as islet amyloid polypeptide (IAPP), amyloid-*β* (A*β*) peptide or *α*-synuclein is responsible both for their aggregation propensity as well as for their strong interface activity.

For several of the amyloidogenic peptides not only interaction with biomembrane surfaces but also membrane penetrating, membrane rupturing, and pore-forming mechanisms are discussed [4]. This aspect of partitioning and penetration through hydrophobic barriers like cell membranes links them to a related class of natural and synthetic peptides, so called cell penetrating peptides (CPP). They have gained importance as a tool for the targeted delivery of non-permeable therapeutics like other peptides, proteins, oligonucleotides or nanoparticles into the cell. [5, 6]

The folding and aggregation equilibria of these systems are usually complex and very sensitive to external factors (ion strength, pH, etc.). In particular, folding patterns are very diverse, spanning from different helical structures to various *β*-structure-rich arrangements [4], and the transitions between these states are coupled to changes in peptide/protein concentration, the presence of initial aggregates as seeds or the presence of membranes, interfaces or surfaces. Aggregate formation appears to occur both in the *α*-helical and in the *β*-sheet state, and in several systems a partially helical structure has been reported prior to fibril formation, while the fibrils themselves typically show *β*-structure-rich regions [4, 7]. For many of these systems an intrinsically disordered state has been found at low concentrations in solution, and it is speculated that this state is an essential intermediate and of great importance for self assembly and aggregation [8].

In particular, very little is known about the low concentration species that are crucial for the early stages of aggregation and that are folding/refolding intermediates. Their molecular state is largely unknown since they are very difficult to detect and characterize experimentally at low concentrations. In addition, their ill-defined fold and their highly dynamic behavior with conformations that remain stable only for short timescales contribute to the challenges in detection. Yet these states are of incredibly high interest as they may well be the actually toxic species in neurodegenerative diseases.

Due to the amphiphilic nature of the peptides/proteins, folding into more defined conformations is observed at interfaces such as the air/water interface or at a biomembrane. In addition to this stabilization of conformations, the interface propensity also leads to an increase in local peptide/protein concentration. Both factors presumably further promote aggregation and fibril formation (see [9–12]). The effect of the interface on the folding process of these—in bulk mostly disordered—peptides/proteins and their aggregation is not understood yet in its entirety [13–16].

Molecular simulation can shed light on the above-described disordered, half-folded, and highly-dynamic states at low concentration. This information can be used to interpret ensemble data from spectroscopic experiments such as NMR, IR or other. In addition, a mechanistic understanding of intrinsically disordered states and the driving forces that trigger folding and aggregation can be obtained. Atomistic insight into unfolded or partially folded intermediates can help to identify the role of different contributions, such as polar and hydrophobic interactions, hydrogen bonding, conformational propensities of amino acid sequences, etc.

**Fig 1:**
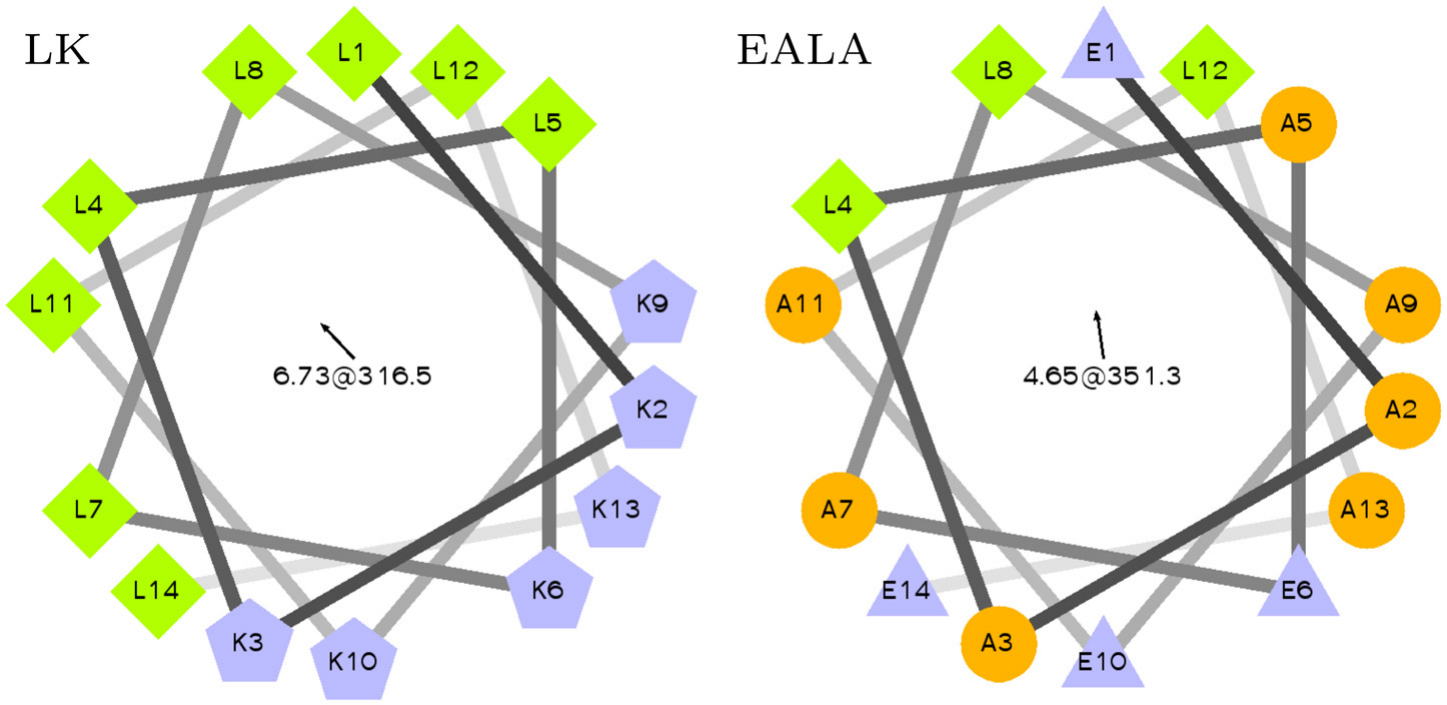
Helical wheel representation of LK and EALA. Both peptides are designed to display secondary amphiphilicity when they adopt the *α*-helix conformation.

In the present study, we use classical atomistic molecular dynamics simulations to investigate the environment-dependent folding equilibrium of two (synthetic) model peptide systems—with a special focus on the influence of soft hydrophobic interfaces and oligomer formation on this equilibrium. The two chosen systems—a cationic model peptide based on LK repeats [17] and EALA, a pH-switchable anionic peptide based on the so-called GALA system [18]—are both designed to display secondary amphiphilicity in the *α*-helical conformation (Fig 1). Both systems are reported to have an affinity to biomembranes and to oligomerize in solution (and in/on the membranes) [17–19]. Peptides composed of leucine and lysine residues have been analyzed in recent studies for their adsorption behavior on hard surfaces with enhanced sampling methods [20], as well as for their role as templates for biomineralization via coarse grained simulations [21]. As we will demonstrate in the course of this paper, both systems display a highly dynamic conformational behavior, with a sizeable amount of intrinsic disorder in solution and an ability to form *α*-helices under certain conditions.

**Fig 2:**
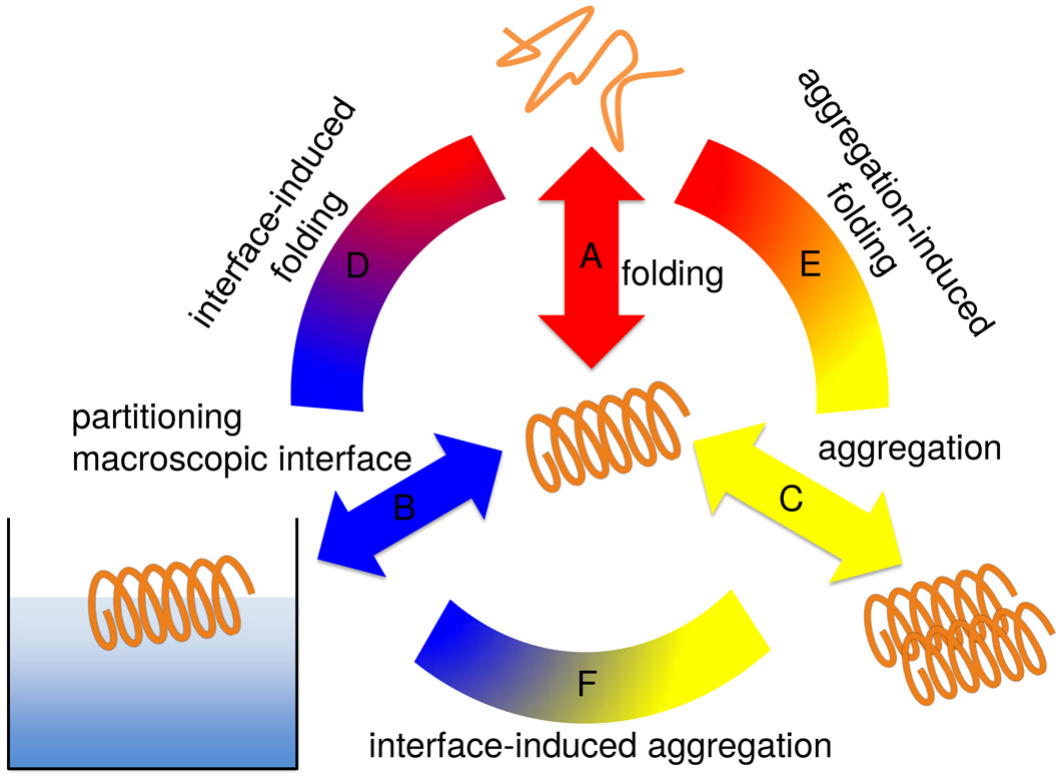
Three separate contributions govern the dynamic conformational equilibrium of an amphiphilic peptide. The folding of the individual molecule in solution (A); the partitioning of hydrophobic/hydrophilic residues induced by macroscopic interfaces (B) or molecular interfaces upon aggregation (C). Combinations of these effects (connecting arcs D, E and F) determine the preferred secondary structure in a given environment.

Thus, our synthetic peptides share the key features of their conformational behavior with natural systems such as *α*-synuclein. With their highly repetitive designed sequences they are good model systems to analyze the contribution of different interactions and driving forces to the coupled equilibria of folding, aggregation and partitioning at (macroscopic) hydrophobic/hydrophilic interfaces (see Fig 2), and to characterize typical microscopic intermediates and pathways. The significant motifs in biological systems such as *α*-synuclein may not be as regular as in the model systems but the general features (such as repeats of charged and hydrophobic elements—see S1 Fig) are comparable.

In the present study we have chosen the most simple representation of a HHI, the (air)vacuum/water interface. Experimentally, the air/water interface is used to study the folding equilibria of various protein systems, and its importance for amyloidogenic intrinsically disordered proteins such as A-*β* or *α*-synuclein has recently been recognized [9, 11, 22, 23]. Air/water interfaces are abundantly present in many experimental systems due to agitation of the samples and they appear to affect fibril formation and growth. From a computational point of view the vacuum/water interface is an ideal soft hydrophobic/hydrophilic model interface that allows to systematically study generic effects and fundamental driving forces.

In the course of the paper we will first characterize the folding equilibrium of individual LKKLLKLLKKLLKL (LK) and EAALAEALAEALAE (EALA) molecules in bulk water (equilibrium A in Fig 2), in particular with respect to potential intrinsic disorder of the individual molecule in bulk, i.e. without a further ordering environmental effect. Then we will subsequently study, how mesoscopic interfaces (in the form of the air/water interface; indicated as connecting arc D in Fig 2), as well as nanoscale interfaces (in the form of a second molecule, as a representative of early stages of oligomerization; indicated as connecting arc E in Fig 2) influence the folding equilibrium of the two molecules. As a last step we will show, how the presence of a HHI affects aggregate formation and possibly further influences the conformational behavior (arc F in Fig 2).

## Results

### Single Peptide in Bulk Water

LK and EALA are both designed to display secondary amphiphilicity [24] in *α*-helix conformation as shown in the helical wheel representation in Fig 1. The hydrophobic/hydrophilic partitioning of the residues in the *α*-helical state plays a major role in determining these peptides’ conformational behavior.

**Fig 3:**
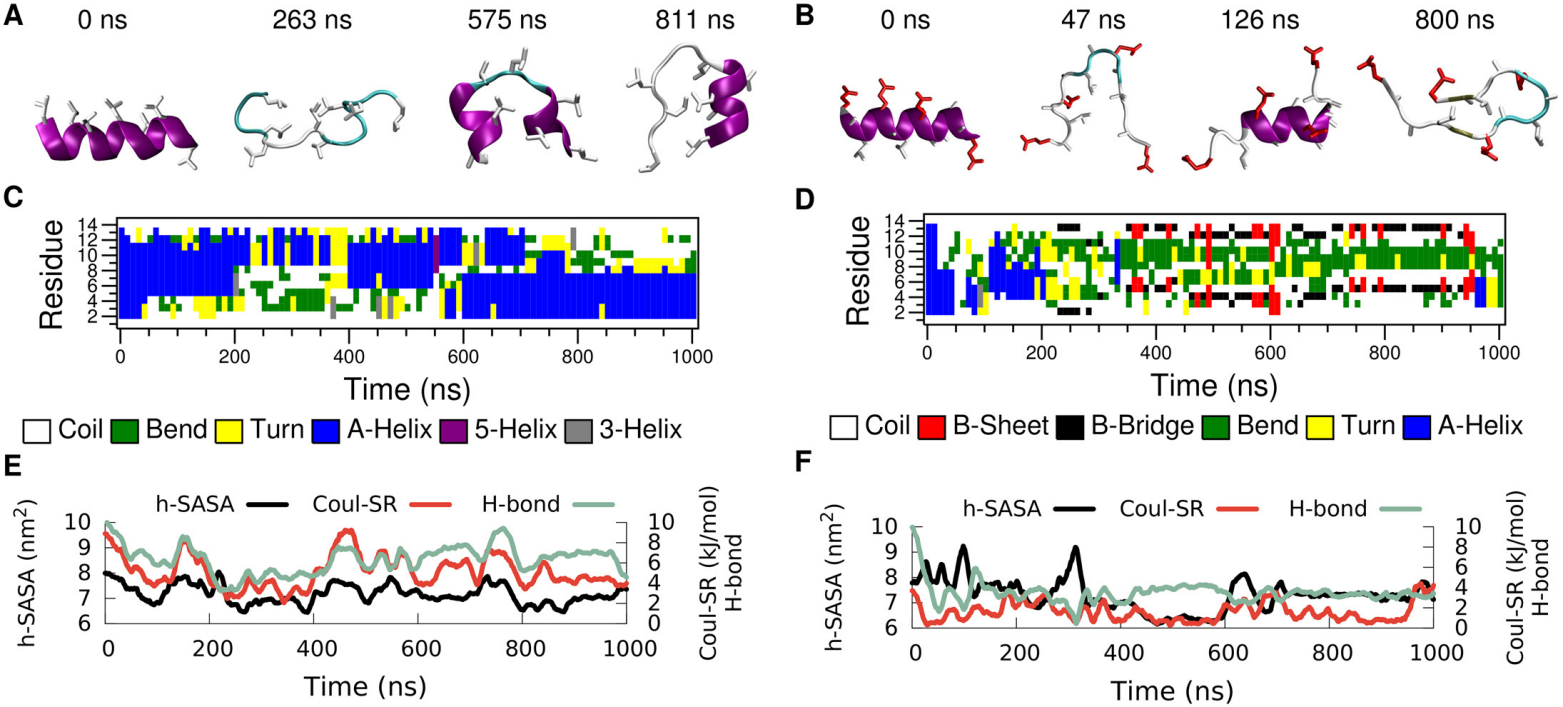
Time evolution of secondary structure for LK (left) and EALA (right) when isolated in bulk water. Snapshots depicting various conformations adopted by these molecules (A and B), DSSP analysis of secondary structure (C and D), SASA for hydrophobic sidechains (h-SASA), number of intra-peptide backbone hydrogen bonds (H-bond) and short-range Coulomb interaction energies (Coul-SR) between the charged groups (E and F) are given for both of the molecules. See Methods section for the color coding and representation of peptides in A and B.

In order to explore the folding-unfolding equilibrium of individual peptides in bulk water (Fig 2 path A) we performed molecular dynamics simulations of LK and EALA. Fig 3 shows the time evolution of the secondary structure for both molecules. The simulations were started from an ideal *α*-helix conformation of the peptides. Analysis of the secondary structural changes via DSSP [25] (Fig 3C and 3D) reveals that for both molecules the *α*-helix conformation is not stable in bulk water. They adopt a variety of different conformations, where a few representative snapshots are shown for LK and EALA in Fig 3A and 3B, respectively. The structural instability of LK and EALA can be assessed based on the competition between three main factors: Coulomb repulsion between the charged sidechains, hydrophobic forces acting on the hydrophobic residues, and backbone-backbone or backbone-sidechain hydrogen bond formation. We discuss the role of these three main factors by comparing their running averages with an ideal *α*-helix conformation in Table 1.

**Table 1:** Average short range Coulomb energy, hydrophobic SASA, and number of intra-mainchain hydrogen bonds for LK, AK and EALA.

peptide	environment	Coul-SR (kJ/mol)	h-SASA (nm^2^)	H-bond
LK	ideal *α*-helix	8.6	8.6	12
	single bulk	5.2 (60%)	7.2 (84%)	6.4 (53%)
	single interface	11.4 (133%)	1.7 (20%)	10.1 (84%)
	dimer bulk	9.0 (105%)	4.9 (57%)	10.4 (87%)
	dimer interface	12.3 (143%)	2.2 (26%)	11.3 (94%)
AK	ideal *α*-helix	10.0	4.6	12
	single bulk	4.9 (49%)	4.4 (96%)	3.8 (32%)
	single interface	11.6 (116%)	1.7 (37%)	11.3 (94%)
	dimer bulk	4.5 (45%)	4.6 (100%)	2.6 (22%)
	dimer interface	–	–	–
EALA	ideal *α*-helix	4.9	7.8	12
	single bulk	1.6 (33%)	7.3 (94%)	3.5 (29%)
	single interface	5.1 (104%)	3.8 (49%)	9.9 (83%)
	dimer bulk	0.6 (12%)	4.8 (62%)	2.8 (23%)
	dimer interface	6.1 (124%)	2.2 (28%)	10.2 (85%)

The running averages are calculated for an ideal *α*-helix in bulk water and under four different setups: a single peptide in bulk water and at the vacuum/water interface, and two peptide molecules in bulk water and at the vacuum/water interface. For all four cases comparison with the ideal *α*-helix is shown as percentages. For the dimers average of the two chains is reported. Running averages are calculated by discarding the equilibration period.

When LK is in ideal *α*-helix conformation all six positively charged lysine sidechains are located on the same side of the helix. The electrostatic self-energy of the peptide can be approximated by the (screened) short range Coulomb repulsion between the charged sidechains (see Methods). Fig 3E and Table 1 show that when the molecule unfolds the Coulomb energy of LK drops to roughly 60% of the value for the *α*-helix. As expected, Coulomb repulsion drives the molecule towards conformations which allow larger distances between the charged sidechains.

With a total of eight leucine residues LK exhibits a rather large hydrophobic surface (8.6 nm^2^), which is completely exposed to water when in ideal *α*-helix conformation in bulk water. Hydrophobic forces drive the molecule towards conformations where the leucine sidechains are less exposed to solvent and form a hydrophobic core. However, this is rather difficult to achieve when LK is isolated on its own. By measuring the SASA for hydrophobic leucine sidechains of LK, one can assess the degree of shielding from water achieved by any particular conformation (Fig 3E). The h-SASA drops on average only by 16% compared to the ideal *α*-helix (Table 1).

The half-*α*-helix conformation, captured with the snapshot at 811 ns, is an attempt to form a hydrophobic core with just a single LK. This conformation is very similar to the TRP-cage structure [26, 27], but it lacks the stability and specificity found for TRP-cage. LK can form a half-*α*-helix structure at either the C- or the N-terminus as observed between 400–550 ns and 700–1000 ns, respectively (see DSSP analysis in Fig 3C). Reduction of the Coulomb repulsion and h-SASA via the loss of the ideal *α*-helix conformation comes at a price: number of backbone hydrogen bonds is reduced by half (Fig 3E and Table 1).

In general all three of these contributions display a positive correlation. For the running averages given in Fig 3E the correlation coefficients of h-SASA with H-bond, h-SASA with Coul-SR, and H-bond with Coul-SR are 0.44, 0.68, and 0.74, respectively. LK peptide is a frustrated molecule, since there is no unique conformation, which is favorable for all three main contributions to free energy, when LK is isolated in bulk water. Experiments at low peptide and chloride concentrations suggest a random conformation for LK. [17] Hence our results at infinite dilution are in agreement with the experimental findings.

EALA, with 4 glutamic acid residues, has a slightly smaller total charge (−4) compared to LK. As for the hydrophobic residues, in comparison to the eight leucine residues of LK, EALA has 3 leucine and 7 alanine residues. Despite the small size of the alanines, due to the increased total number of hydrophobic residues, the h-SASA value for EALA is only slightly smaller than LK, 7.8 *nm* ^2^, for the ideal *α*-helix conformation LK (Table 1).

Similar to LK, EALA (with deprotonated glutamic acid sidechains at pH 7) lacks a well defined equilibrium structure in bulk water (see Fig 3B and 3D). However, EALA shows considerably less tendency towards *α*-helix conformation. Qualitatively the individual secondary structure elements found for EALA appear to have shorter lifetimes compared to LK.

Given that under acidic conditions, when the sidechains are protonated, half GALA (the system from which EALA had been derived, see also S1 Fig) folds into an *α*-helix in bulk water [28], electrostatic repulsion can be listed as the dominant driving force in the unfolding of the helix. Upon unfolding the short range Coulomb interactions are reduced to 33% of the ideal *α*-helix conformation (Table 1). h-SASA is not significantly changed, and hydrogen bonds are down to 29% of the ideal *α*-helix conformation. Interestingly, no strong correlation between h-SASA, hydrogen bonds and electrostatic repulsion is observed (the correlation coefficient for h-SASA with H-bond, h-SASA with Coul-SR, and H-bond with Coul-SR are -0.27, 0.14, and 0.16, respectively.

Of course, MD results from a single simulation (even when it is one microsecond long) do not reveal the full picture, the system is not in full thermodynamic equilibrium and we are far away from having sampled states and transitions repeatedly and exhaustively. To get a better impression of the degree of sampling (or the lack thereof), we have performed additional simulations for the LK peptide with different initial velocities (results are given in S2 Fig). In these additional MD runs, the sequence of conformations adopted by LK differed considerably, however the main conclusions did not change: None of the conformational changes, which take place over several hundred nanoseconds, lead to a single stable conformation of the molecule. Hence, both LK and EALA can be characterized as peptides with a high degree of intrinsic disorder in bulk aqueous solution. However, it is important to note that these molecules do not behave like a random coil. LK displays a higher propensity towards *α*-helical conformations, while EALA mostly adopts loop-like structures which transiently change into *β*-hairpins.

### Single Peptide at the Vacuum/Water Interface

**Fig 4:**
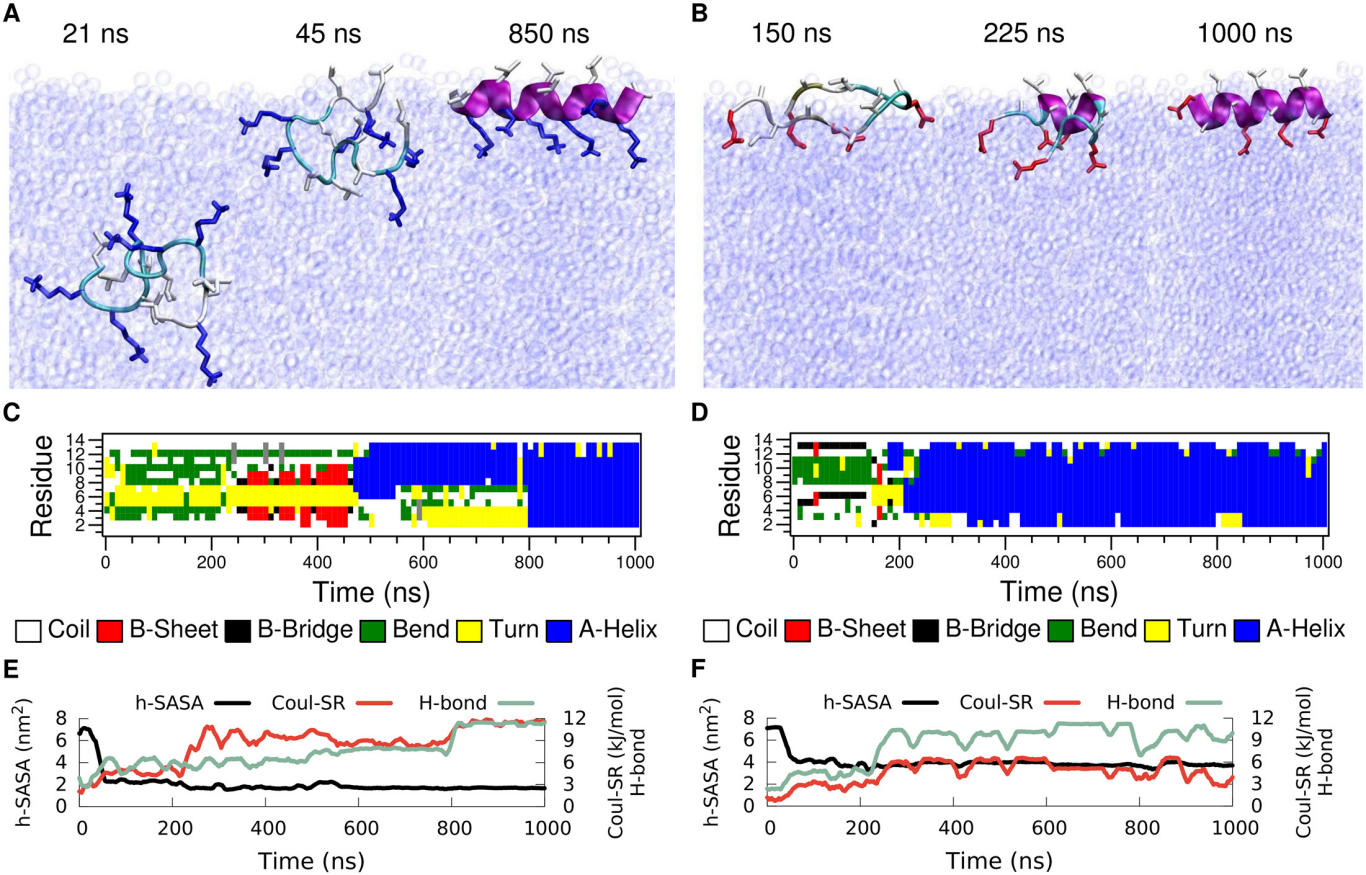
Simulations of a single peptide at the vacuum/water interface for LK (left) and EALA (right). Simulations are started with random conformations at the center of the water slab. Upon adsorption of peptides (after 45 ns for LK and 50 ns for EALA) partitioning of hydrophobic/hydrophilic residues and folding into the *α*-helix structure (after 800 ns for LK and 350 ns for EALA) is observed. Snapshots depicting the adsorption and adoption of the *α*-helix structure (A and B) and the DSSP analysis of the secondary structure evolution (C and D) are shown for both molecules. SASA, intra-peptide backbone hydrogen bonds and short-range Coulomb interaction energies between the charge groups (E and F) display the partitioning of hydrophobic/hydrophilic residues and secondary structure formation in the presence of the interface.

In this section we investigate LK and EALA at the vacuum/water interface, in particular we show the drastic effect that this interface has on their conformational preferences (Fig 2 arc D). As in the previous section we first focus on the behavior of a *single* peptide molecule. Initially, the molecules were positioned at the center of the water phase in a random conformation (exemplary shown for LK in Fig 4A snapshot at 21 ns). After a short time (45 ns for LK and 50 ns for EALA) both molecules diffuse to the interface. In the particular simulations shown here, the peptides still lacked a well defined secondary structure when they reached the interface (Fig 4C and 4D) and folded subsequently. Since in bulk water the peptides adopt a large variety of transient conformations, molecules can in principle also make the first contact with the interface with a partially formed secondary structure.

The first structural change observed at the interface is the partitioning of the hydrophobic and hydrophilic residues as marked by the sharp drop in h-SASA (Fig 4E and 4F). Note that h-SASA calculation is performed such that only the surface of the hydrophobic residues that are actually in contact with the solvent is taken into account (see Methods section). Coupled with this reduction in h-SASA, the initial signs of secondary structure formation can be seen via an increase in the number of intra-peptide backbone hydrogen bonds (Fig 4E and 4F). At the same time, the partitioning effect leads to a locally increased density of charged sidechain groups in the water phase resulting in an increased electrostatic repulsion (green curve) which, however, is not strong enough to prevent folding.

Upon partitioning LK adopts a *β*-hairpin structure which lasts up to 475 ns, at which point a transition to the *α*-helix conformation starts. This half *α*-helix structure persists for more than 300 ns, which finally transforms into a full *α*-helix. Similarly, after going through a series of conformational changes, EALA adopts a full *α*-helix conformation. Separately, for both molecules we simulated systems where the molecule was directly placed at the interface in an *α*-helical conformation(S3 Fig). In these simulations *α*-helix structure remained completely stable for more than one microsecond for both peptides. Hence, we can safely conclude that in the presence of a hydrophobic/hydrophilic interface both LK and EALA adopt an *α*-helix as their preferred conformation. Note that the transition to the *α*-helical conformation does not significantly reduce the h-SASA for both peptides compared to the other states at the interface (black lines in Fig 4E and 4F). The hydrophobic sidechains are already desolvated as a result of partitioning, therefore the h-SASA is not strongly affected by folding. The remaining driving force for folding into an *α*-helix is the formation of hydrogen bonds. For those parts of the peptide that are not exposed to water, intramolecular (*α*-helical) hydrogen bond formation will be very favorable. Comparing the total values of h-SASA and electrostatic interactions between LK and EALA once the helix is formed at the interface, for EALA both the favorable reduction of h-SASA upon partitioning as well as the unfavorable Coulomb repulsion upon helix formation are smaller than for LK (Table 1). An analysis of the distribution of hydrophobic and hydrophilic sidechains at the interface can be found in S4 Fig. The data clearly show the preferential orientation of the LK and EALA helices as expected.

**Fig 5:**
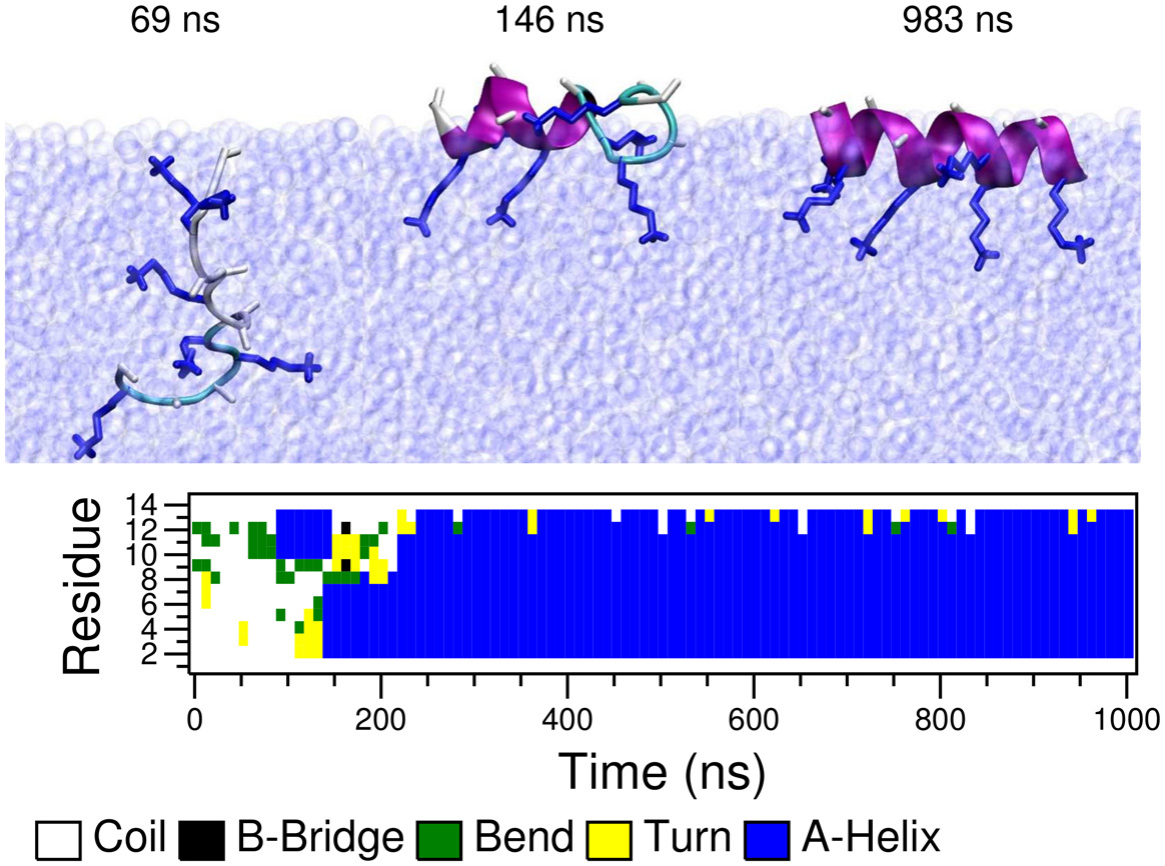
Time evolution of secondary structure of a single AK (in-silico mutated form of LK) peptide at the vacuum/water interface. Initially the peptide is submerged in bulk water and moves to the interface in 80 ns and remains there for the rest of the simulation.

In order to better understand the contribution of the hydrophobic sidechains to the observed folding and partitioning equilibria, we have performed an *in silico* mutation of the leucine residues to alanine in LK, resulting in an alanine-lysine peptide (from now on denoted as AK). Fig 5 shows timeline and snapshots of the secondary structure formation of a single AK in the presence of the vacuum/water interface. Similar to LK and EALA, AK peptide, which was initially placed at the center of the water layer with a random conformation, moved to the interface in a short time (approx. 80 ns) and subsequently folded into *α*-helix. The distribution of alanine and lysine sidechains with respect to the interface are shown in S4 Fig. Notably, the decrease in sidechain hydrophobicity apparently neither prevents the partitioning nor the folding at the interface.

In summary, the vacuum/water interface dictates a strong partitioning of the hydrophobic and hydrophilic residues in all three peptides considered. Once the partitioning takes place–since all three molecules are designed to display secondary amphiphilicity in the *α*-helix conformation–complete folding and stabilization of secondary structure is observed. All the peptides diffuse to the interface in an unfolded conformation and fold subsequently, with AK and EALA folding significantly faster than LK (see Figs 4 and 5). This observation can be explained by the fact that folding at the interface requires intermediate conformations where hydrophobic sidechains are (re-)solvated in water and hydrophilic sidechains are temporarily desolvated. This former step for example is more unfavorable for leucine than for alanine sidechains which may affect the height of the transition barriers in the folding process. The observations suggest that the folding times at the interface may be related to the hydrophobic moment [29] of the peptides, with LK being the slowest one to fold with the highest hydrophobic dipole. Further data collected from a variety of sequences will be needed to support this hypothesis.

### Two Peptide Molecules in Bulk Water

As demonstrated in the previous sections, the conformation of these intrinsically disordered peptides strongly depends on the environment. In bulk water both LK and EALA display a variety of secondary structures, which also include partial *α*-helices. However, in the presence of a vacuum/water interface, all alternative secondary structures, except the full *α*-helix are completely eliminated, supporting the conformation-selection & population-shift hypothesis for intrinsically disordered systems [30, 31]. Interfaces are not limited to macroscopic ones. In the cell environment or in an experimental setup at finite concentration, proteins and peptides are surrounded by several other molecules. These surrounding molecules also present an interface, which we will refer to as a molecular interface. These nanoscale interfaces differ from the macroscopic ones (such as the vacuum/water interface) in two important ways. First, in terms of their size, they are of molecular scale and therefore force the guest molecule to fit to this area. Second, they are not permanent but rather transient structures and their stability is strongly influenced by the guest molecule.

**Fig 6:**
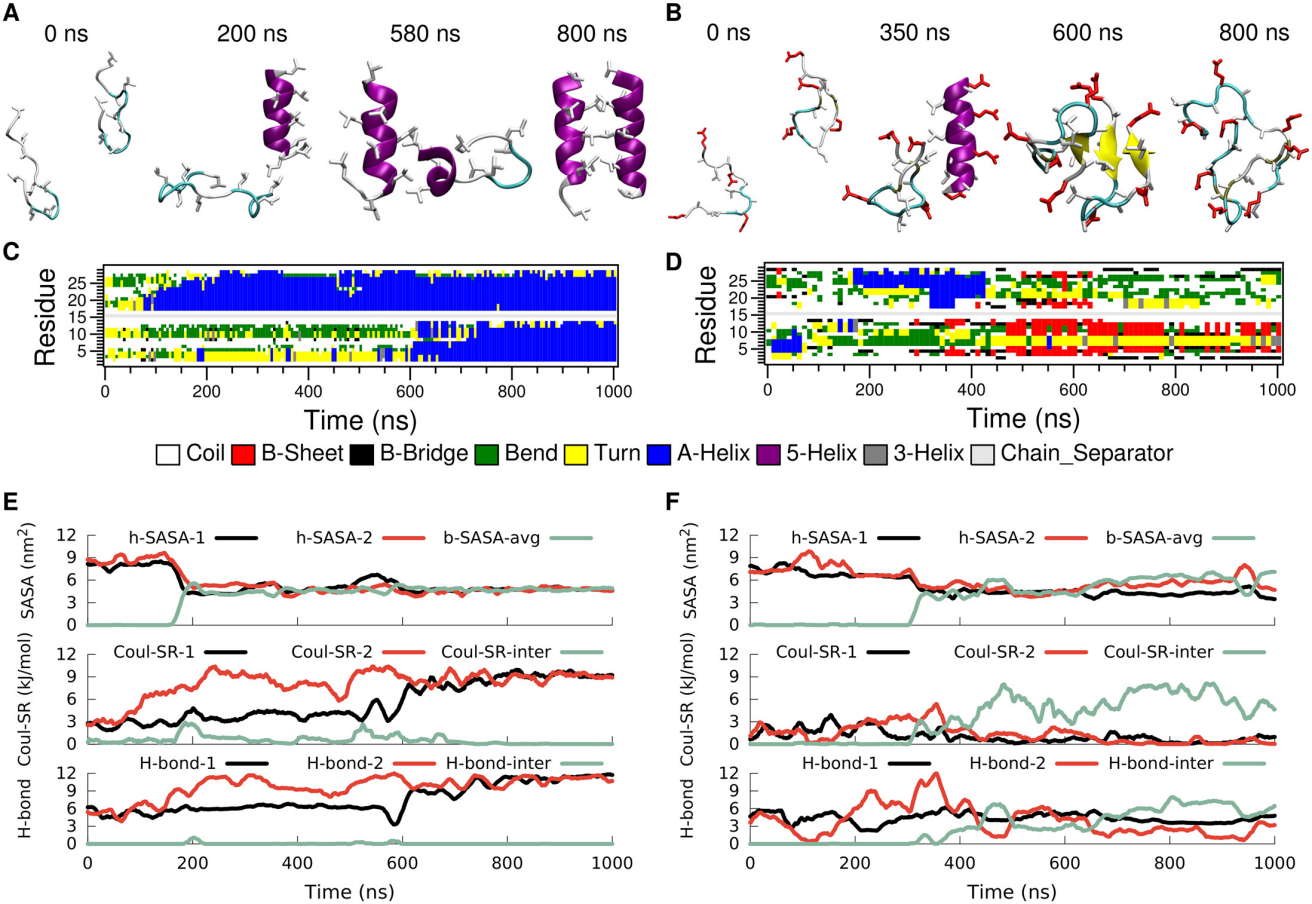
Folding and association of a pair of LK (left) and EALA (right) peptides in bulk water. Snapshots illustrating the aggregation process (A and B), DSSP secondary structure analysis (C and D), SASA, short range Coulombic interaction energies between the charged groups and the number of intra and inter-peptide backbone hydrogen bonds (E and F) are displayed as a function of simulation time. Association of peptides take place at 180 ns for LK and 300 ns for EALA, which can be observed via the sharp drop in SASA.

As a first example we have studied systems with two identical molecules in bulk water to see how the presence of such a molecular interface can affect the folding equilibrium (Fig 2 arc E). Simulations for both LK and EALA were started with two molecules, each in random conformations and separated by several water layers (see Fig 6A and 6B). Snapshots depicting the association of the peptides and time series of the secondary structures, the h-SASA and buried SASA values, the short range Coulomb energy between the charged sidechains, and the number of backbone hydrogen bonds are presented in Fig 6 for the course of one microsecond simulation.

As shown before, neither LK nor EALA single peptides in bulk water adopt a unique conformation. Now, with two peptides, the behavior of LK and EALA is qualitatively different from each other. For LK (Fig 6 left) the DSSP data show that initially one of the molecules starts to form an *α*-helix at around 80 ns (Fig 6C). Initial contact between the two separate LK molecules is made at around 200 ns via a pair of leucine sidechains (Fig 6A). From this initial contact on, a hydrophobic interface between the two molecules is formed rather rapidly. It appears that the intrinsically disordered nature of LK accelerates the aggregation process in good correspondence with a fly-casting mechanism [32], where initial contact is established via extension of unfolded tails (see S5 Fig, S1 and S2 Videos). Although the kinetic effect due to the fly-casting mechanism is reported to be less than 2-fold [32], it may nevertheless be significant for an intrinsically disordered system where multiple pathways exist, and small kinetic advantages may tip the balance, in particular in typical non-equilibrium experimental situations.

The formation of the hydrophobic interface can be observed in the SASA plot (Fig 6E). The h-SASA of each peptide chain declines rapidly at around 180 ns and the buried SASA corresponding to the contact area of the peptides increases. This overall behavior persists for a timespan of approximately 400 ns. Finally, at a simulation time of approximately 600 ns, also the second molecule folds into an *α*-helix. The increase of sidechain Coulomb repulsion within each individual molecule upon *α*-helix formation which is perfectly correlated with the respective increase in the number of backbone hydrogen bonds (Fig 6E) is completely analogous to the single peptide folding simulations discussed earlier. At the same time the arrangement of the two helices relative to each other allows for an inter-molecular separation of the charged NH_3_ groups such that the inter-molecular Coulomb repulsion approaches zero (dropping lower than in the earlier stages of the aggregation between 100 and 600 ns where one of the peptides is still unfolded). This shows that the dimer interface consists mainly of hydrophobic residues. The fully formed *α*-helices remain stable for the rest of the simulation.

A separate run where the LK peptides were initially setup as aggregated *α*-helices and remain stable throughout, supports these observations (S6 Fig). Hence, LK molecules can act as hydrophobic/hydrophilic molecular surfaces for each other, and the matching interface stabilizes the designed *α*-helix conformation. Comparing the molecular interface with the macroscopic one for LK, one can see that the h-SASA is only reduced to 57% of the value of an ideal *α*-helix that is fully exposed to water. In comparison the vacuum/water interface reduces the h-SASA to 20%. This suggests that the dimerization does not yet lead to a perfect shielding of hydrophobic sidechains from water, i.e. the aggregation is not necessarily limited to two peptides. This is in good agreement with the experimental findings that LK forms tetramers in aqueous solution [17]. Preliminary simulations of a preaggregated LK-tetramer have shown indeed, that this aggregate is stable in solution (see S7 Fig). Further analysis to determine the equilibrium aggregate size and free energy profile are under way.

The stabilization of a specific conformation in the aggregate—in the present case the *α*-helix—due to contact with a hydrophobic surface presented by neighboring peptides can also be observed in other systems. For example in diphenylalanine in solution, a conformational transition is observed upon aggregation: in bulk water the molecule adopts a trans-like state, but the molecular interface provided by the surrounding molecules in an aggregate triggers the conformational transition to a cis-like state so that the hydrophobic phenylalanine side chains can get in contact with hydrophobic patches on the surface [33].

The picture is rather different for EALA. At first, similar to LK, the two molecules get in contact at roughly 300 ns and form a hydrophobic interface with their hydrophobic side chains which leads to a reduction in h-SASA. However, unlike LK, EALA molecules do not form or maintain *α*-helical conformations in the aggregate. In the dimer simulation shown in Fig 6 (right panel), one of the EALA molecules displays a *β*-hairpin structure, whereas the second molecule does not adopt a well defined secondary structure (Fig 6D). Even if one starts with both EALA molecules in *α*-helix conformation the picture does not change (S8 Fig): the molecules merge, however multiple times in the course of this 2 microsecond long simulation one of the two helices unfolds and adopts different secondary structure elements, such as turn or coil structures.

One key difference for EALA is that the glutamic acid residues play a dual role: due to their charge they repel the other EALA molecule while they can at the same time make contact with the backbone of the neighboring molecule because of their hydrogen bonding capacity. Indeed, the inter-molecular hydrogen bonds (Fig 6F) show that the two EALA molecules are connected via hydrogen bonds in addition to hydrophobic contacts. In the case of LK no inter-peptide hydrogen bond formation is observed (Fig 6E) and the dimer is held together purely by hydrophobic attraction. It should be noted that these sidechain-backbone hydrogen bonds potentially destabilize the *α*-helices, and predominantly show up for EALA dimers without *α*-helical elements (S8 Fig).

**Fig 7:**
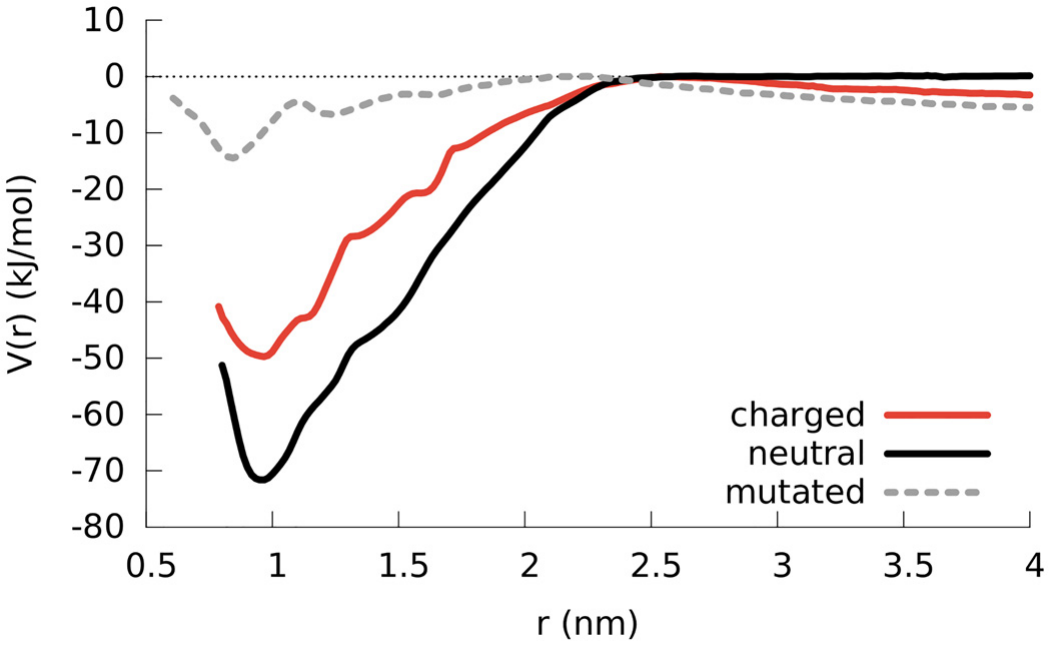
PMF results comparing the separation of a LK dimer in bulk water for the cases where lysine residues are positively charged, lysine residues are neutral and the leucine residues are in silico mutated to alanine residues. The curves are shifted so that the maximum points are zero. The distance refers to the distance between the center of mass of backbone atoms of the peptides. For the mutated AK dimer, when the peptides are in contact they maintain their helical structures. However when they are separated or when they make a loose contact the *α*-helical structure is not conserved.

To quantify the strength of the dimerization, we employed umbrella sampling and computed the potential of mean force (PMF) as a function of the distance between two LK peptides. Fig 7 compares different PMFs to identify contributions from electrostatic and hydrophobic interactions: between two normal, i.e. charged, LK molecules (red line), between two neutral LK molecules where the lysines hold no charge (black line) and for the previously mentioned AK molecules (with leucine mutated to alanine; dashed grey line). The Coulombic repulsion between the positively charged lysine residues decreases the propensity of aggregation as evident from the approximately 20 kJ/mol difference between the charged and neutral dimers at the PMF minimum (red and black curves, respectively). When the peptides are completely separated (approximately above 2 nm), one observes a repulsion which can be seen from the slope in the PMF curves of charged LK and AK dimers. The fact that this slope does not exist for the PMF of the neutral LK dimer confirms that the repulsion is of electrostatic origin, resulting from the lysine charges. This repulsion acts as a barrier against aggregation where the probability of overcoming it can be increased by unfolded peptides, initiating contact through hydrophobic residues. The hydrophobic attraction between the peptides for the AK dimer is smaller compared to the LK dimer because of the reduced volume of hydrophobic groups, supporting the hypothesis that the aggregation is driven by the formation of the hydrophobic core. Due to the reduced hydrophobic attraction in the AK dimer, the peptides unfold easily, yielding different PMF curves for the folded and the unfolded cases, which makes it harder to achieve convergence in a reasonable amount of time. As a result, the block analysis of the PMF yields different curves for different blocks (see S9 Fig). However, the poor convergence does not change the fact that the aggregation of AK is significantly reduced compared to LK. This is further confirmed by unconstrained simulations of two AK molecules (see S10 Fig) which neither fold into *α*-helices nor aggregate (as can be seen from the values for buried SASA and inter-peptide hydrogen bonds). Even when the simulation starts with a pre-aggregated *α*-helical AK dimer, the molecular interface is not sufficient to maintain the helical aggregate (see S11 Fig) where, the peptides unfold and after one microsecond, the aggregate is weakly held by inter-chain hydrogen bonds. These data show that the mutation from leucine to alanine completely removes aggregation propensity from the LK peptides, as well as the tendency to form transient partial helical elements. In this sense AK is the most drastically disordered peptide investigated here. Remarkably it does form *α*-helices at the air/water interface, though.

Given the previously observed instability of secondary structures for EALA in solution and in aggregation (which hinders convergence), no PMF calculation were performed for two EALA molecules.

### Two Peptide Molecules at the Interface

Considering the strong influence of the interface on the conformation of peptides, we finally investigated the combined effect: two peptides at the vacuum/water interface (Fig 2 arc F). As we had observed that the interface induces the *α*-helix conformation in both molecules, the simulations were started from pre-folded *α*-helices. For EALA, the two peptides were aligned at the interface, initially well-separated from each other by several layers of water molecules. For LK, the peptides were initially aggregated, but this starting configuration does not introduce any bias on the system, as the molecules display multiple aggregation/dissociation events during the simulation.

**Fig 8:**
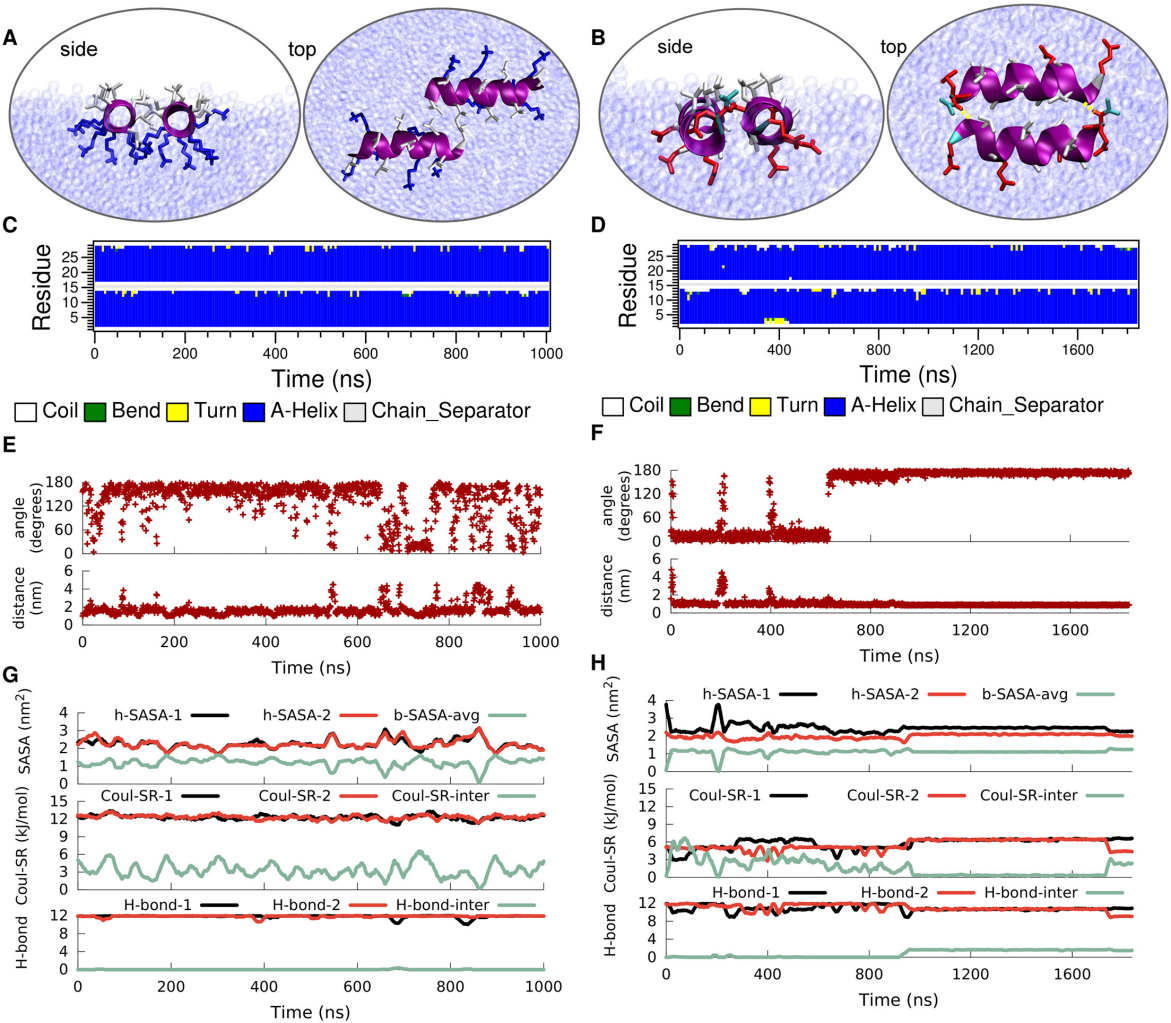
Time evolution of the secondary structure of a dimer of LK (left) and EALA (right) at the vacuum/water interface. Typical snapshots when the peptides are associated at the interface (A and B), DSSP structural analysis (C and D), angle between the helix axis for the peptides and center-to-center distance (E and F), the h-SASA for each peptide along with the buried SASA for the whole peptide, the inter and intra molecular short-range Coulomb energies and the number of inter- and intra-molecular hydrogen bonds (G and H) are shown in figure.

Both LK peptides retain their *α*-helical structure for the entire simulation as characterized by DSSP analysis (Fig 8C). In bulk water a strong attraction was observed between dimers for both LK and EALA, such that once aggregated the peptides did not fully separate again for the course of the simulation. For LK, though, the presence of the interface weakens this inter-peptide attraction compared to the association strength in bulk water. Fig 8A is a representative snapshot which shows that the LK molecules prefer to have only a partial alignment with the other peptide. In fact the dimer frequently falls apart and regroups as quantified by the angle and the center of mass distance between the peptides (Fig 8E). Distance-angle correlation plots (see S12 Fig) reflect the contrast between dimer interactions in bulk and at the interface. In bulk water, once aggregated, the two peptides maintain their alignment and distance very strictly as evident from a single peak in the distance-angle correlation plot, whereas at the interface the correlation plot is much more scattered. Conformations corresponding to the three most populated states for LK dimer are also given in S12 Fig, and among them the most preferred state is where the peptides only partially align with each other.

This reduction in dimer stability for LK at the vacuum/water interface compared to bulk is a direct consequence of the disruption of the hydrophobic core. In bulk water hydrophobic leucine residues of the two peptides face each other and form a hydrophobic contact interface, whereas at the vacuum/water interface the leucine sidechains preferentially extend into vacuum instead of towards the leucine sidechains of the neighboring peptide. Furthermore, the alignment of the sidechains of both peptides at the vacuum/water interface causes the charged lysines to come into closer contact compared to the dimer in bulk water (see snapshot in Fig 8A), resulting in unfavorable electrostatic repulsion. Therefore, the inter-peptide short range Coulomb energy (Fig 8G) for the dimer at the interface is higher compared to the one for the dimer in bulk water (Fig 6E). Both of the peptides yield similar h-SASA values to the single LK at the interface due to the identical stability of the *α*-helix. One can follow the aggregation of the dimers from both the inter-peptide distance (Fig 8E) and buried SASA (Fig 8G) which are inversely correlated. Moreover, the molecules do not make any inter-molecular hydrogen bonds.

Once again the picture is rather different for EALA. Under the strong influence of the interface both EALA peptides retain their *α*-helical conformation (Fig 8D). In this conformation they strongly associate with each other by forming leucine-leucine contacts at the interface. Unlike LK, for EALA charged glutamic acid residues do not appear to prevent such an alignment, presumably because they are differently distributed on the peptide surface. Differently from LK, the dimeric state of EALA at the interface appears to be more deeply submerged into the water phase compared to a single EALA molecule (S4 Fig). This suggests that dimerization and mutual shielding of hydrophobic sidechains leads to an overall structure where other parts of the molecule can be more favorably solvated. Initially EALA peptides orient in a parallel fashion, which switches to the anti-parallel alignment at 600 ns. Upon switching an additional stabilizing factor comes in to play: hydrogen bonds at the two ends of the aggregate.

Fig 8H shows the intra and inter-peptide hydrogen bonding for EALA. Inter-peptide hydrogen bonds that appear after ca. 900 ns, at the cost of intra-peptide ones, lead to the locking-in of the antiparallel orientation. Same trade-off can be observed for the electrostatic repulsion where the inter-chain value is minimized while the intra-chain repulsion is increased. The buried SASA is in agreement with the enforced interaction and additionally shows that the orientational changes, such as flipping of the peptide between anti-parallel and parallel orientations, are possible while the peptides stay in contact. The h-SASA of the aggregate is strongly reduced by a factor of 2 compared to a single peptide at the interface and keeps the hydrophobic area of the dimer at the same level as a single molecule. While h-SASA and Coulomb interaction describe the less explicit or long range interactions, the formation of the inter-chain hydrogen bonds locks in the angle and distance between the peptides for the rest of the simulation (Fig 8B, 8F, and 8H).

## Discussion

In this paper, we have investigated the influence of interfaces on the conformational behavior of two synthetic peptides, EALA and LK, along with its alanine mutant AK, with built-in secondary *α*-helical amphiphilicity. First, we have characterized the behavior of individual molecules in bulk water and found that they are neither random coils nor fully formed *α*-helices, but adopt multiple conformations and secondary structure elements with short lifetimes on the order of 100 ns or less. These peptides would—as isolated molecules in bulk water—be experimentally characterized as partially or even fully intrinsically disordered. Often it is not quite clear, to which extent intrinsically disordered systems are composed of truly random coils and to which extent they are a broad ensemble of partially folded structures with very short lifetimes. This time- and ensemble-averaging makes a clear experimental characterization extremely difficult. Our results suggest that intrinsically disordered peptides are not necessarily always fully disordered. They do populate a variety of more or less well-defined conformational states, but none of them are dominant and long-lived enough to stabilize the system into a unique fold.

A change in environmental conditions typically changes the relative depths of the free-energy minima corresponding to these ensemble structures. This possibly explains why for many intrinsically disordered polypeptides certain environmental triggers induce the formation of distinct structures. The formation of *β*-sheet-rich amyloid fibrils upon aggregation of IDPs such as A-*β* or *α*-synuclein can be given as examples.

One important class of external stimuli that induce structure formation related to secondary amphiphilicity are interfaces—both macroscopic as well as molecular ones. We first investigated the conformational behavior of LK and EALA under the influence of the macroscopic vacuum/water interface. This interface leads to a strong partitioning of hydrophobic/hydrophilic residues, which enforces a unique conformation by stabilizing the *α*-helical form for both model systems. In the case of the LK peptide, the bulk water ensemble is already dominated by conformations where a large fraction of the residues are in an *α*-helical state. On the contrary, for EALA the probability of structures with *α*-helical elements in the bulk water ensemble is low (but non-zero). Thus, the shift towards fully formed *α*-helices for both systems supports a conformational-selection & population-shift hypothesis, where the partitioning of the side chains at the interface shifts the existing equilibria and enhances the population of the *α*-helical structures that are (at least partially) already present.

In their response to molecular interfaces presented by an aggregation partner in bulk water the peptide systems differ considerably. For LK, the molecular interface stabilizes the *α*-helical state, even already at a dimer level. On the other hand, EALA dimers adopt a broad distribution of transient secondary structural elements, including (a very small fraction of) *α*-helical elements as well as *β*-sheet structures. For AK the further reduced hydrophobicity of the sidechains destabilizes the dimer state entirely. Our MD simulations and PMF calculations have shown that the differences between LK, EALA and AK can be linked to the peptides’ inherent *α*-helical tendencies in bulk water, as well as to their different balance of hydrophobic forces, electrostatic repulsion, and intra- and intermolecular hydrogen-bonding.

The simulations also allow an interesting glimpse on to the early stages of dimer formation, when the two molecules make their first contact. Not surprisingly, the first contact is established via the extended tail of one of the molecules in its disordered state as proposed in the fly-casting mechanism. This gives rise to the speculation, that a certain degree of disorder is kinetically advantageous for aggregation of these peptide systems, since it makes random encounters more likely. One might speculate that it is not entirely coincidental that so many amyloid forming peptides are intrinsically disordered in solution since (*i*) they are conformationally ambiguous and (*ii*) disorder may enhance aggregation.

We find that the peptides’ aggregation behavior is also strongly affected by presence or absence of a macroscopic interface. While the LK peptides aggregate less strongly at the vacuum/water interface compared to bulk water, two EALA molecules form very stable *α*-helical dimers at the vacuum/water interface. The different behavior of EALA can be linked to the distribution of surface charge and hydrophobicity, and again, strong intermolecular hydrogen bonds further stabilizes the dimer.

In the present work we have focused on the qualitative analysis of the interface effect on conformational behavior of model peptides. Enhanced sampling techniques could potentially be utilized to obtain a more quantitative description of the conformational space of such molecules. However, this becomes extremely challenging for processes that do have an important diffusive component such as dimer formation. In the future it would be promising to augment these atomistic simulations with additional ones on a more coarse grained (CG) level of description, giving access to biologically relevant time and length scales. However, it is essential for such CG models to correctly reflect the environment dependent conformational preference of the molecules as seen in the present work.

## Methods

### Setup

The amino acid sequences for LK and EALA are Ace-(LKKLLKL)_2_-Nme and Ace-(EAALAEALAEALAE)-Nme, respectively. All simulations were performed at neutral pH where the lysine sidechains in LK were protonated, and the glutamic acid sidechains in EALA were deprotonated. The systems were neutralized by six chloride and four sodium ions for LK and EALA respectively.

### Simulations

Molecular dynamics simulations were conducted using GROMACS version 4.5.6 [34] with the leap-frog integrator employing the GROMOS 54a7 force-field [35] and SPC/E water [36]. All bonds were constrained with LINCS algorithm [37] to enable a time-step of 2 fs. Coulomb interactions were calculated by particle mesh Ewald (PME) [38] method with a 1 nm cut-off. Lennard Jones interactions were calculated with a twin-range cut-off scheme of 1.0 and 1.4 nm with a neighbor list update at every 10 steps.

All systems were energy minimized with steepest-descent algorithm for 2000 steps, followed by three consecutive simulations for equilibration (100 ps each) where the heavy atoms, the backbone atoms and the C_*α*_ atoms were constrained. This was followed by a 100 ps simulation for further equilibration. The actual simulations for data collection were at least one *μs* long.

Simulations in bulk water and interface were performed at 298 K by using the Velocity-rescaling algorithm [39] with a coupling time of 1 ps. The bulk water systems were simulated in an isobaric-isothermal ensemble where the pressure was set to 1 atm using isotropic Berendsen pressure coupling [40] with a pressure relaxation time of 1 ps for both the solvent and the peptide. The isothermal compressibility of 4.5×10^−5^ bar^−1^ which corresponds to that of pure water was used for the system.

The simulations for the single peptide in bulk water had 2919 and 2965 water molecules for LK and EALA respectively with cubic boxes of dimension of 4.5 nm on the average. The dimer simulation for LK in bulk water was started in a rhombic dodecahedron box of dimensions 7.27 nm in x and y and 5.14 nm in z-dimension with 8882 water molecules. After the LK molecules merge, to accelerate the simulation, the system size was reduced to 2984 molecules in a box size of 5.12 × 5.12 × 3.62 nm. In order to verify that the box size does not change the outcome the original simulation with the larger box size was extended until folding (S13 Fig). In case of EALA, the simulation box had the dimensions of 8.03 × 8.03 × 5.68 nm and 12108 water molecules.

Vacuum/water interface simulations were performed with systems with cubic water slabs that have two interfaces to a vacuum slab in z-direction. These simulations were performed in the canonical (NVT) ensemble to maintain the shape of the box. For the single peptides the box sizes were 5.16 × 5.16 × 16.00 nm and 6.0348 × 6.0348 × 18.1043 nm, with 4467 and 7268 water molecules for LK and EALA respectively. The size of the vacuum in z-dimension was chosen to be twice the size of the z-dimension of the water slab. The dimer simulations at the vacuum/water interface contained 10258 (LK) and 10331 (EALA) water molecules in rectangular boxes with average dimensions of 6.78 × 6.78 × 20.35 nm.

The potential of mean force (PMF) calculations in Fig 7 were performed by the umbrella sampling method [41] where a harmonic potential was applied to the distance between the center of mass of backbone atoms of the peptides. Initially one of the peptides is pulled away from the other with a rate of 0.01 nm/ps and at intervals snapshots were obtained which, upon energy minimization and equilibration, form the initial configurations of the umbrella windows. A force constant of 1000 kJ mol^−1^ nm^−2^ was used to restrain the distance between the peptides. Each window for the charged and neutral cases was sampled for 200 ns whereas the in silico mutated case was run for 300 ns. Weighted histogram analysis [42] was applied to obtain the PMF curves using the g_wham tool [43] of GROMACS. The convergence of the PMF curves was checked with the block analysis method.

### Analysis

Solvent accessible surface area (SASA) calculations were performed with a probe size of 0.24 nm using the g_sas tool of GROMACS. The relatively large probe was chosen to avoid inclusion of cavities in bulk water for the SASA calculations at the interface (see below). With few exceptions (which are separately indicated in the text) we report the SASA of the hydrophobic (alanine and leucine) sidechains of the peptides, denoted as h-SASA. Hence, for a single peptide in bulk water the g_sas tool is used by specifying the whole peptide as calculation group (i.e. the specification of what is considered solute and what is solvent), but restricting the output to only the surface of the hydrophobic sidechains. At the vacuum/water interface the calculations are done in two steps. First, by using the peptide as calculation and the hydrophobic sidechains as output group, the whole contact area of the hydrophobic sidechains is calculated. Next, by using water and peptide as calculation group and the hydrophobic sidechains as output group, the contact area of the hydrophobic sidechains facing vacuum is calculated. Taking the difference between the two values yields the h-SASA excluding the area exposed to vacuum. For the dimer state of peptides both peptides are given as calculation group, but only the hydrophobic sidechains of one of the peptides is given as output group. Finally, for two peptides at the interface, the same two step calculation is performed to calculate the h-SASA of the hydrophobic sidechains that are actually in contact with the solvent as described above for the single molecule at the interface. In addition, we compute also the buried (total) SASA between the two peptides. This is the difference between the sum of the SASA values of the individual peptides and the SASA of the aggregate (in the case of the interface simulation again accounting for the fact that part of the surface is exposed to the vacuum).

Effective electrostatic energies of the solvated peptides were calculated using the “rerun” option of GROMACS’ mdrun tool: the electrostatic interaction between the protein charges was re-calculated using a cutoff scheme with a relative permittivity of 80 to account for the screening by water. In Figs 3, 4, 6 the short range Coulomb interaction energies between the CE-NH_3_ groups at the end of the lysine sidechains and the COO groups at the end of the glutamic acid sidechains are considered for LK and EALA respectively. Hydrogen bond analysis was performed using the g_hbond tool of GROMACS using a 30° cutoff for the acceptor-donor-hydrogen angle and a 3.5 Å cutoff for the distance between donor and acceptor. The reported inter-chain hydrogen bonds are between the main chain atoms unless otherwise stated. SASA, hydrogen bonds and the electrostatic energies are presented as running averages over 2000 frames with a data step size of 10 ps throughout the paper. The ideal *α*-helix data in Table 1 are the average values from a 100 ns simulation where the single peptide is initially an ideal *α*-helix with the backbone atoms position restrained throughout the simulation.

All visualizations were produced by VMD [44]. In Figs 3, 4, 6, and 8 snapshots of the backbone of the peptide are shown in cartoon representation of VMD, and colored based on the secondary structure. The secondary structures were assigned by the STRIDE algorithm [45]. The sidechains of the aminoacid residues are drawn in licorice representation and colored according to polarity, where the lysine is blue, the leucine and alanine are white and the glutamic acid is red. In Figs 3 and 6 the lysine sidechains and the water molecules are hidden for clarity. Helical wheels in Fig 1 were obtained using the “Helical Wheel Projection” tool [46].

## Supporting Information

S1 Fig Sequences of LK, EALA and GALA and comparison with *α*-synuclein. The figure shows the sequences of the peptides studied in the present paper (LK and EALA), and for reference, also the experimental model peptide GALA is shown from which the EALA model peptide had been derived. GALA/1-30 refers to the full GALA sequence whereas the GALA05/1-16 represents a shortened version as studied in [18]. The difference between GALA and EALA lies in the replacement of the tryptophane and histidine which are used as spectroscopic labels. GALA had been simulated at two different protonation states in [18]: At neutral pH the histidine sidechain is neutral and glutamic acid sidechains are deprotonated, the C-terminus is deprotonated and the N-terminus is protonated. At low pH, the histidine, glutamic acid sidechains as well as the C- and N- termini are protonated. The figure also shows a comparison with the part of the sequence of *α*-synuclein where an *α*-helical state is formed upon membrane binding which relies on amphiphilic repeats. The alignment shows similarities in the hydrophobic profiles, and even though the sequence similarity between *α*-synuclein and EALA and LK is not high, there are repeating motifs in the hydrophobicity profile showing the secondary amphiphilicity. The colors range from blue to red for the hydrophilic to hydrophobic residues respectively.
(TIFF)

S2 Fig DSSP analysis for the additional runs of single LK peptide in bulk water. In order to test the dependence on the initial conditions we have run three additional MD simulations with different initial velocities. In two of these runs LK displayed a high *α*-helix content in its secondary structure, whereas in the second one the predominant structure is observed to be a *β*-hairpin. These additional runs demonstrate that LK is an intrinsically disordered peptide, as it does not have a preferred secondary structure on its own in bulk water. The percentages of residues with defined secondary structures: 44% *α*-helix, 26% coil, and 17% bend & turn (top); 8% *α*-helix, 25% *β*-sheet, 36% coil, and 20% bend & turn, (middle); 41% *α*-helix, 24% coil, and 19% bend & turn (bottom); and finally 44% *α*-helix, 34% coil, 20% bend & turn for the simulation shown in the manuscript.
(TIFF)

S3 Fig Single peptide at the vacuum/water interface with *α*-helical starting conformation. DSSP analysis of time evolution of the secondary structure of a single LK (top) and EALA (bottom) at the vacuum/water interface color coded for each secondary structure.
(TIFF)

S4 FigAtom density plots comparing single peptides (solid lines) and dimers (dashed lines) at the vacuum/water interface: LK (top) and EALA (middle).The plot at the bottom compares the single LK with the single AK in the same environment. Vertical gray lines indicate the position of the interface where the interface is determined as the point as the water density is half of that of bulk water. Density plots are shown for backbone atoms (black), hydrophobic sidechains (red), and hydrophilic sidechains (green) separately. The calculated groups are as follows: iso-propyl group at the end of leucine sidechain (labelled as LEU(ip)), nitrogen atom at the end of lysine sidechain (labelled as NZ) and CO_2_ group at the end of glutamic acid sidechain (labelled as GLU(${\text{CO}}_{2}^{-}$). Slices for computing densities are taken every 1 Å. The snapshot at the bottom is of a single AK at the vacuum/interface where similar to LK, the lysine sidechains (blue) extend into water and the alanines face vacuum.(TIFF)

S5 Fig Screenshot for the movies showing fly-casting mechanism for LK and EALA dimers in bulk water. The snapshots depict the initial contact formation via unfolded chain-ends. For LK, the leucine residues are colored red and lysine residues are colored blue, for EALA the same coloring scheme in the manuscript is applied here. The ACE caps are shown with yellow CPK representation.
(TIFF)

S6 Fig DSSP analysis of the simulation of two LK molecules initially in *α*-helix conformation and pre-assembled into a dimer. The *α*-helix conformation and the aggregate remain stable for 1 *μ*s, in agreement with the simulation results presented for a pair of LK molecules in bulk water with random starting conformations.
(TIFF)

S7 Fig Pre-aggregated LK tetramer in bulk water. The peptides remain aggregated and *α*-helical throughout the simulation time of 1 microseconds. The snapshot shows that the aggregate forms a hydrophobic core dehydrating the leucine sidechains. The ACE caps are shown with yellow CPK representation.
(TIFF)

S8 Fig Additional EALA dimer simulation in bulk water where the peptides are initially *α*-helical and separated. Time evolution of secondary structure (top) and h-SASA, short range Coulombic interaction energies and the number of hydrogen bonds of the main chain (solid) along with the hydrogen bonds between the sidechain and the main chain (dashed) are shown (bottom). As can be seen from the buried SASA (green curve) the peptides aggregate approximately in 50 ns. Although the dimer remains stable, the peptides do not maintain the initial full *α*-helical secondary structure unlike the LK dimer in bulk water. After unfolding, the peptide can make hydrogen bonds between the sidechain and the main chain at the cost of the ones between the main chains of the molecules. This is visible after 1800 ns and it illustrates the counterplay between the different inter and intra molecular driving forces acting in the folding and aggregation processes. This conformation increases both intra-peptide and inter-peptide electrostatic repulsion. The inter-peptide hydrogen bonding compensates for the intra-molecular hydrogen bonding as well as the electrostatic repulsion, while for SASA the effect is less pronounced.
(TIFF)

S9 Fig The block analysis for the PMF of separating an AK dimer. Because of the unfolding of the AK peptides the PMF blocks for the first 150 ns (purple) and the second 150 ns (yellow) yield different values. Although this behavior prevents proper sampling in a reasonable amount of time, it does not change the result that the aggregation energy of AK dimers is less than LK. Entropic correction is applied to the PMF curves and they are shifted so that the PMF equals zero at the moment of total separation at 2.1 nm.
(TIFF)

S10 Fig Two AK peptides in bulk water initially separated and unfolded. DSSP analysis of secondary structure (top), SASA of the sidechains of hydrophobic residues and short-range Coulomb interaction energies between the charge groups are given. Similar to the EALA and unlike LK dimer in bulk water, the AK peptides remain unfolded and do not show any stable secondary structure. However, in contrast to EALA, the stronger charge repulsion (six charged lysine residues in AK versus four charged glutamic acid residues in EALA) together with the reduced hydrophobic attraction does not allow a stable aggregate as seen from the buried SASA.
(TIFF)

S11 Fig Two AK peptides in bulk water pre-aggregated and pre-folded into *α*-helix. The snapshots of the initial (left) and the final structures (top), time evolution of secondary structure (middle), hydrophobic SASA of the sidechains, short-range Coulomb interaction energies between the charge groups and number of hydrogen bonds (bottom) are given. Unlike the LK dimer the peptides unfold after approximately 350 ns. Although the peptides are unfolded, they are still in contact after one microsecond. The final structure is not stable and it is held weakly by inter-chain hydrogen bonds. As a result of the separation of the inter-residue charge groups the intra-Coulomb short range energies decrease, where at the same time the inter-dimer ones increase. The final h-SASA values are higher than the initial folded values, because the peptides cannot dehydrate their hydrophobic residues through a stable hydrophobic core.
(TIFF)

S12 Fig Orientational analysis for dimer simulations. Inter-dimer distance vs inter-dimer angle correlation plots for LK (a and c) and EALA (b and d) in bulk water (top) and at the air/water interface (middle). Part e shows the representative snapshots of the most populated dimer arrangements of LK peptides at the air/water interface (c). The DSSP plots can be seen in Fig 6 for the ones in bulk water (top), and in Fig 8 for the simulations at the vacuum/water interface (middle). The correlation plot for the LK dimer in bulk water contains the data after the peptides are aggregated and folded (first 800 ns of the simulation shown in part a was discarded).
(TIFF)

S13 Fig Extension of the simulation of initially unfolded and separated LK peptides in bulk water. After the initial 500 ns the box size of the simulation in the manuscript was reduced to match the smaller aggregate size. The outcome is similar to the one presented in the manuscript as well as the results.
(TIFF)

S1 Video Fly-casting mechanism observed for two LK dimers in bulk water. The leucine residues are colored red and lysine residues are colored blue. The ACE caps are shown with yellow CPK representation.
(MP4)

S2 Video Fly-casting mechanism observed for two EALA dimers in bulk water. The ACE caps are shown with yellow CPK representation.
(MP4)
